# Tracking progress towards sustainable development goal 3.2 in Somalia using time series models: a comparative forecasting analysis

**DOI:** 10.1186/s13031-026-00808-y

**Published:** 2026-06-03

**Authors:** Khadar Mowlid Abdi, Abdirisak Mohamed Moumin, Hibo Abdilahi Omer, Saralees Nadarajah, Abdisalam Hassan Muse

**Affiliations:** 1 Research and Innovation Center, Amoud University, Borama, 25263 Somalia; 2 Faculty of Science and Humanities, School of Postgraduate Studies and Research (SPGSR), Amoud University, Borama, 25263 Somalia; 3 Faculty of Business and Economics, Amoud University, Borama, 25263 Somalia; 4 Department of Mathematics, University of Manchester, Manchester, M13 9PL UK; 5 School of Dentistry, Amoud University, Borama, 25263 Somalia

**Keywords:** ARIMA, ANN, Hybrid, Forecasting, Infant mortality rate

## Abstract

**Background:**

High infant mortality rate (IMR) is a critical public health indicator. Understanding its trajectory is vital for policymaking, particularly in Somalia, where rates remain elevated. This study aimed to model and forecast Somalia’s IMR to identify the most accurate method, providing data-driven insights for public health strategy.

**Objectives:**

The study aimed to formulate time series models, propose hybrid models, and rigorously compare their performance in forecasting IMR.

**Methods:**

This quantitative time-series analysis utilized annual World Bank data for Somalia from 1950 to 2022. Three models were developed: Autoregressive Integrated Moving Average (ARIMA), Artificial Neural Network (ANN), and a hybrid ARIMA-ANN model. Performance was evaluated using Root Mean Square Error (RMSE) and Symmetric Mean Absolute Percentage Error (sMAPE) on a test set spanning 2013 to 2022.

**Results:**

Upon achieving stationarity via first-order differencing, the ARIMA (1, 1, 3) model proved most effective. It demonstrated superior accuracy with an RMSE of 0.85, significantly outperforming the ANN (RMSE 1.04) and hybrid models (RMSE 3.01). Validated ARIMA forecasts project a continued, gradual decline in IMR from 59.02 deaths per 1,000 live births in 2023 to 40.37 in 2032.

**Conclusion:**

The ARIMA model offers the most reliable framework for forecasting Somalia’s infant mortality rate based on historical data.

## Introduction

Infant mortality rate (IMR) reflects the health status of both mothers and newborns, being especially responsive to structural factors including socioeconomic progress and fundamental living standards. IMR is defined as the probability of a child dying before their first birthday. Infant mortality rate is observed as essential national indicator of health because it’s specifically sensitive to structural factors such as socioeconomic development and basic living conditions [1]. Globally, the infant mortality rate has decreased significantly from 8.8 million over the past [2]. Between 1990 and 2017, the IMR decreased from 65 fatalities per 1000 live births to 29 deaths per 1000 live birth [3]. When compared to the third Sustainable Development Goal (SDG-3),whose objective is to ensure healthy lifestyles and promote the well-being of all at all ages” by 2030, the IMR in Sub-Saharan Africa (SSA) countries dropped from 182 to 58 fatalities per 1000 live births between 1990 and 2017 [4].

Globally, Sub-Saharan Africa holds the highest IMR at 81 per 1000 live births [5]. In Rwanda, over the past 10 years, infant mortality has declined gradually, from 86 deaths per 1,000 live births in 2005 to 62 deaths per 1,000 live births in 2007-08, 50 deaths per 1,000 live births in 2010, and 32 deaths per 1,000 live births in 2014-15 [6]. In Rwanda, a study utilizing machine learning on the 2014–15 Demographic Health Survey dataset was conducted to track progress toward Sustainable Development Goal 3 (SDG-3).The IMR in Nigeria is estimated at 70 per 1000 live births implying that 1 in 15 live births in Nigeria die before their first birthday [7].

Additionally, the IMR in Kenya was 31.87 per 1,000 livebirths in 2019, reducing by 32.97% from 1995 [8]. Ethiopian government has persisted infant mortality as a major issue with the nation’s healthcare system. Ethiopia is one of the countries with a high IMR in Africa. Infant mortality in Ethiopia has decreased from 97 per 1000 live births in 2000 to 48 per 1000 live births in 2016, with significant regional and national variations [9].

Somalia presents a unique and challenging context for health forecasting due to the prolonged civil conflict that began in 1991, which resulted in the collapse of central government institutions, including the national statistical bureau and public health infrastructure. For decades, the country has lacked a unified system for vital registration, leading to significant data scarcity. The high Infant Mortality Rate in Somalia is not merely a health metric but a reflection of these systemic fragilities, including limited access to maternal healthcare, nutritional insecurity, and recurring climate-induced droughts. Therefore, developing robust forecasting models using reconstructed secondary data (such as World Bank estimates) is critical for filling these information gaps and guiding resource allocation in a post-conflict reconstruction setting.

The study adopted the Sustainable Development Goals established by United Nations (2015),particularly (SDG-3), since it focuses on to ensure healthy lives and promote the well- being for everyone at every age by 2030 because of the core aim of SDG-3 is to diminish a high prevalence of infant mortality. The motivation for this study is drawn from the realization that knowledge of reliable and accurate forecasts of infant mortality rate is both necessary and important for the planning of suitable intervention programs and preventive measures for the reduction of infant mortality in Somalia. Results from several studies have shown that the ARIMA model if carefully selected can be fitted to the time series data of single variable to forecast, with better accuracy, the future values in the series. Research has consistently highlighted the complex interplay of factors like maternal health, nutrition, and access to healthcare in influencing infant mortality [10].

Furthermore, advancements in data analytics and machine learning are increasingly being applied to improve the precision of health outcome predictions, including IMR [10].The ongoing challenge of reducing infant mortality in developing nations underscores the critical need for robust forecasting models to guide policy and resource allocation [7].Therefore, this study aims to contribute to this body of knowledge by evaluating and comparing different time-series models for forecasting IMR in Somalia, a region where such data-driven insights are particularly valuable. The selection of appropriate statistical models for forecasting public health indicators is crucial for effective health planning and intervention strategies [11]. This research also considers the impact of socio-economic factors on IMR trends, which are often significant in low-income countries [12].

Based on the dataset from World Bank, 1950–2022, high IMR in Somalia has reported for the last 73 years. However, it had declined the last years since IMR for Somalia in 2022 was 64.097 deaths per 1000 live births, a 1.99% decline from 2021, also in 2021 was 65.399 deaths per 1000 live births, a 1.95% decline from 2020 but in 2020 was 66.702 deaths per 1000 live births, a 1.91% decline from 2019 but in 2018 was 69.307 per 1000 live births Despite, IMR for Somalia is still high. Therefore, this research aimed to apply ARIMA, ANN, and hybrid models to forecast the IMR in Somalia using World Bank data from 1950 to 2022.

### Statement of novelty and model justification

While time-series forecasting is well-established in public health, its application to the Somali context remains a critical gap in the literature. Somalia presents a unique demographic landscape characterized by prolonged civil conflict, the collapse of the central state in 1991, and fragile health infrastructure. Previous studies frequently aggregate Somalia into broader Sub-Saharan African regional analyses, masking the localized volatility in its health data. This study addresses this gap by providing a localized, comparative forecasting analysis to track Somalia’s progress toward SDG 3.2.

Furthermore, model selection is highly dependent on data architecture. We selected ARIMA, Artificial Neural Networks (ANN), and a Hybrid ARIMA-ANN model over alternatives like Long Short-Term Memory (LSTM) or Facebook Prophet. Deep learning models like LSTM require vast amounts of high-frequency data to train effectively without overfitting, while Prophet is optimized for daily or weekly seasonal data. Given our dataset relies on annual historical records (73 observations), ARIMA is optimally suited to capture the strong linear trends inherent in demographic mortality rates, while the Neural Network Autoregression (NNAR) architecture effectively maps localized non-linearities without the massive data requirements of deeper networks.

## Materials and methods

### Study setting and design

This study employed a quantitative, longitudinal time-series analysis design to model and forecast the infant mortality rate (IMR) in Somalia. The setting is the nation of Somalia, with data representing the entire country at an annual frequency. The design is based on the application and comparison of three distinct forecasting models—ARIMA, ANN, and a hybrid ARIMA-ANN model—to historical secondary data. The research follows the Box-Jenkins methodology for ARIMA Model Identification, estimation, and diagnostic checking.

### Study population and sampling procedure

The study population consists of a complete set of 73 annual data points for the Infant Mortality Rate in Somalia, spanning the period from 1950 to 2022. The data is secondary and was obtained from the World Bank data base.

### Data reliability, preprocessing, and structural breaks

Utilizing long-term historical data (1950–2022) in a conflict-affected nation poses inherent challenges. During the height of the Somali civil war (particularly 1991–2000), vital registration systems collapsed. Consequently, data points from this era rely on retrospective household surveys (such as MICS and SHDS) and UN Inter-agency Group for Child Mortality Estimation interpolations. While these represent the best available estimates, they introduce a degree of smoothing.Given this history of conflict, it is critical to mathematically test for structural breaks in the demographic data. A Zivot-Andrews unit root test was applied to the training dataset. The test successfully identified a potential structural break at position 39, which corresponds to the year 1989. This mathematically aligns with the onset of the Somali civil war and the collapse of the central government, justifying the need for differencing in our models to account for this historical shock. Following standard practice, the dataset was partitioned into a training set (1950–2012, approx. 85%) to capture these historical patterns, and a testing set (2013–2022, approx. 15%) to validate the model’s performance on recent, SDG-era trends.

#### Data partitioning

The dataset was partitioned using a standard time-series sampling procedure.

#### Training set

The first 63 observations (1950–2012) were used to train and build the models (see Figure 2).

#### Test set

The final 10 observations (2013–2022) were held out to evaluate the predictive accuracy (see Figure 3).

#### Study variables

This was a univariate study focusing on a single primary variable.

#### Dependent variable

The Infant Mortality Rate (IMR) for Somalia. This is a quantitative, continuous variable defined as the number of infant deaths under one year of age per 1,000 live births.

#### Independent variable

The independent variable is Time, measured in annual increments. For the forecasting models, lagged values of the IMR were used as predictors to forecast future values.

### ARIMA model specification

Model selection for the ARIMA analysis was rigorously based on minimizing the Akaike Information Criterion (AIC) and the Bayesian Information Criterion (BIC) to ensure the most parsimonious model fit.

#### Advanced evaluation metrics

To rigorously evaluate predictive accuracy and address limitations of standard error metrics (RMSE/SMAPE), we expanded our criteria to include Willmott’s Index of Agreement (WI), Absolute Percentage Bias (APB), and the Explained Variance Score (EVS). Willmott’s Index provides a standardized measure of prediction error bounded between 0 and 1, where 1 indicates perfect agreement. To determine if the differences in predictive accuracy between the chosen models were statistically significant, we employed the Diebold-Mariano test.

### Artificial neural network (ANN) architecture

For the Artificial Neural Network (ANN) modeling, a feed-forward neural network architecture was employed using the nnetar function in R. The data was pre-processed using min-max scaling to normalize values between 0 and 1. The network utilized a sigmoid activation function in the hidden layers and a linear activation function in the output layer. The training algorithm used was resilient backpropagation, with the number of hidden nodes (k) automatically selected based on forecast accuracy.

### Hybrid (ARIMA-ANN) model” section

The hybrid methodology employs an additive framework defined by Yt = Lt+Nt. First, the ARIMA model is used to generate the linear forecast component (Lt). The residuals (ϵ_*t*_) from this linear model, calculated as ϵt = Yt−Lt, are assumed to contain the non-linear relationships that ARIMA cannot capture. An ANN model is then trained using these residuals as the target variable to predict the non-linear component (Nt). The final forecast is the summation of the ARIMA prediction and the ANN predicted residuals.

### Ethical considerations

This study utilized publicly available, aggregated secondary data from the World Bank database. As the data is anonymized and public, ethical clearance was not required.”

## Results

**Fig. 1:**
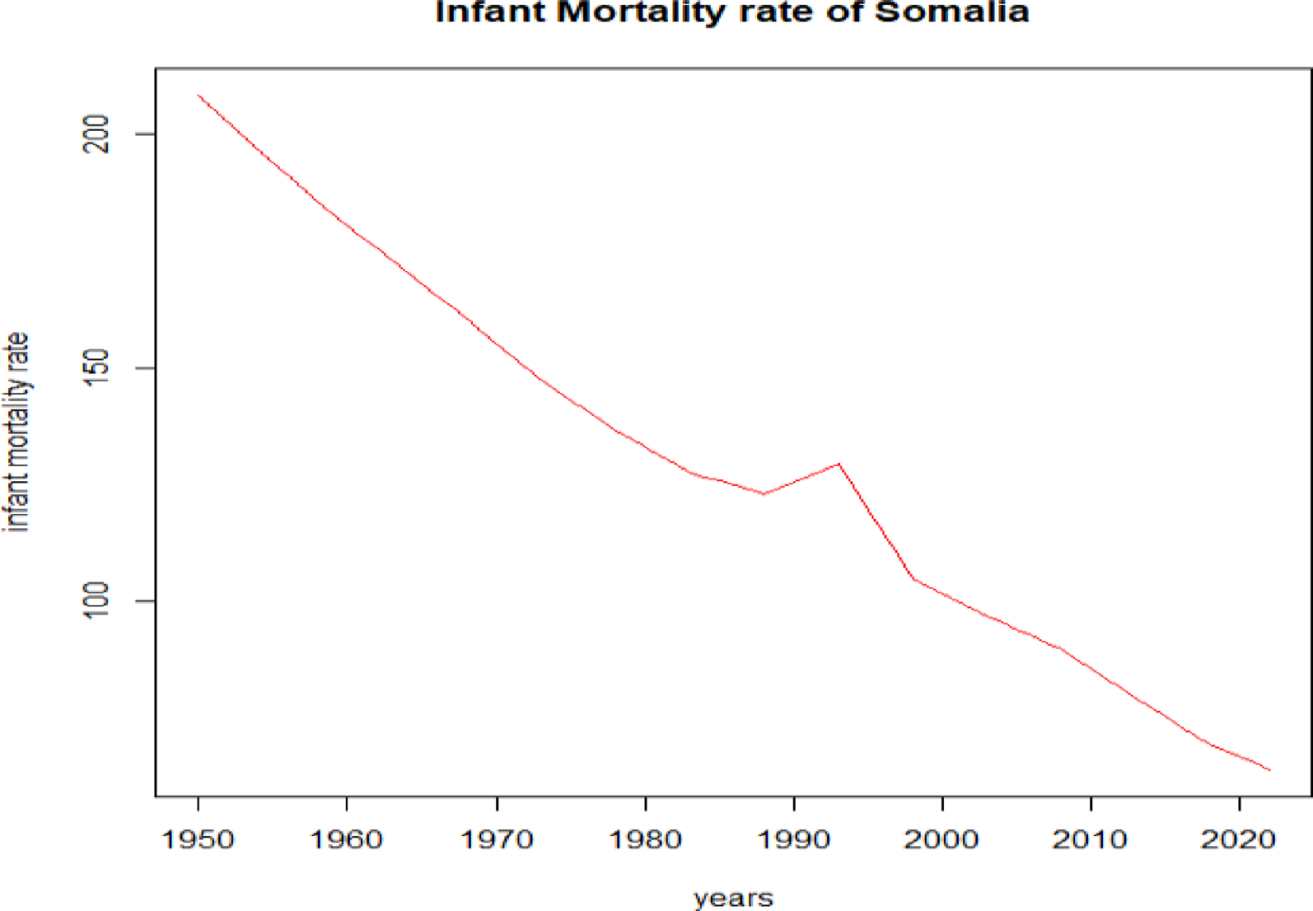
Infant mortality rate data of Somalia from 1950-2022

**Fig. 2:**
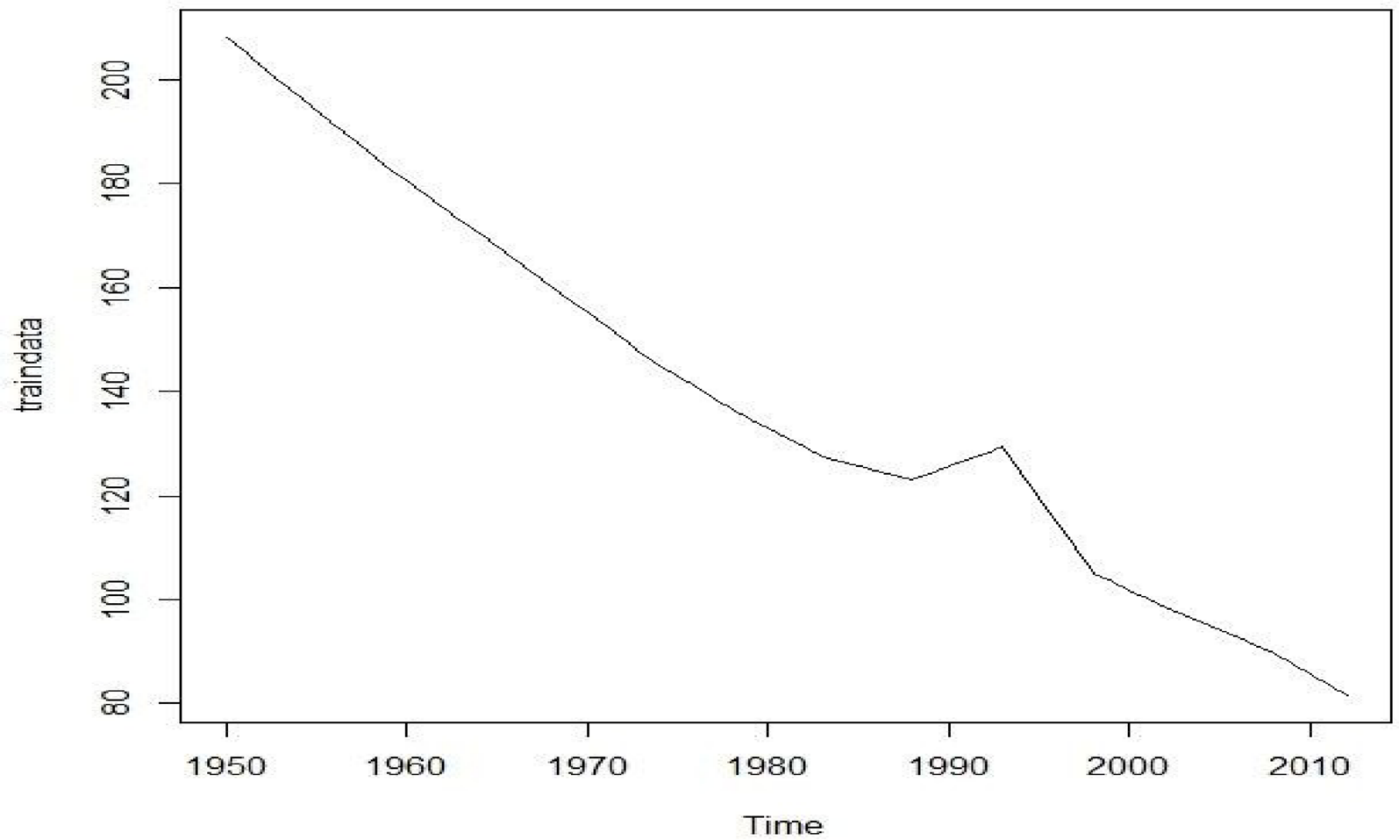
Train data from 1950 to 2012 of infant mortality rate in Somalia and shows that IMR was decreasing

**Fig. 3:**
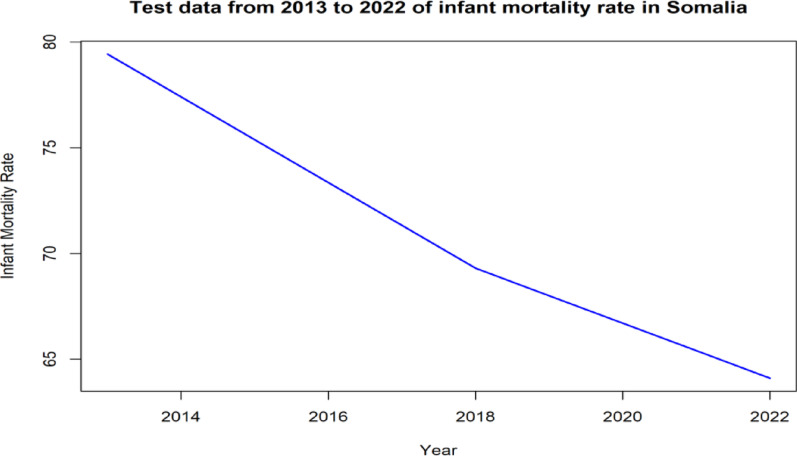
Test data from 2013 to 2022 of infant mortality rate in Somalia

There were a total of 73 observations. The data has a mean of 129.60 and a median of 126.90. The standard deviation of the training data was 40.56, indicating high variability in the trend (see Table 1 and Figs. 1, 2 and 3).

**Table 1:** Descriptive Statistics of the Infant Mortality rate data of Somalia from 1950-2022

Statistic	Value
N	73
Mean	129.60
Median	126.90
10% trimmed mean	128.50
Geometric mean	123.10
Skewness	0.197
Kurtosis	−0.947
Minimum	64.10
Maximum	208.30
Range	144.20
1 st Quartile (Q1)	95.52
3rd Quartile (Q3)	160.30
Standard deviation	40.55
Geometric standard deviation	1.39
Interquartile range	64.78
Median absolute deviation	48.63
Coefficient of variation	0.31

### ARIMA Modeling

#### Differencing

We first investigated differencing to ensure stationarity. The results of the Augmented Dickey-Fuller (ADF) test yielded a p-value of 0.03018, indicating that the data is stationary at the first difference. To avoid bias, we examined the ARIMA (p,1,q) model and utilized R’s auto.arima() function, which selected ARIMA (1,1,3) in Table 2.

**Table 2:** Augmented dickey-fuller Test for the first difference infant mortality rate data

	Augmented Dickey-Fuller	Lag order	p-value
Value	−3.7234	3	0.03018

The Phillips-Perron test results are detailed in Table 3.

**Table 3:** Phillips-perron test for the first difference infant mortality rate data

	Phillips-perron	Lag order	p-value
**Value**	−19.156	3	0.03192

Trend, ACF, and PACF plots for the first differencing infant mortality rate data.

**Fig. 4:**
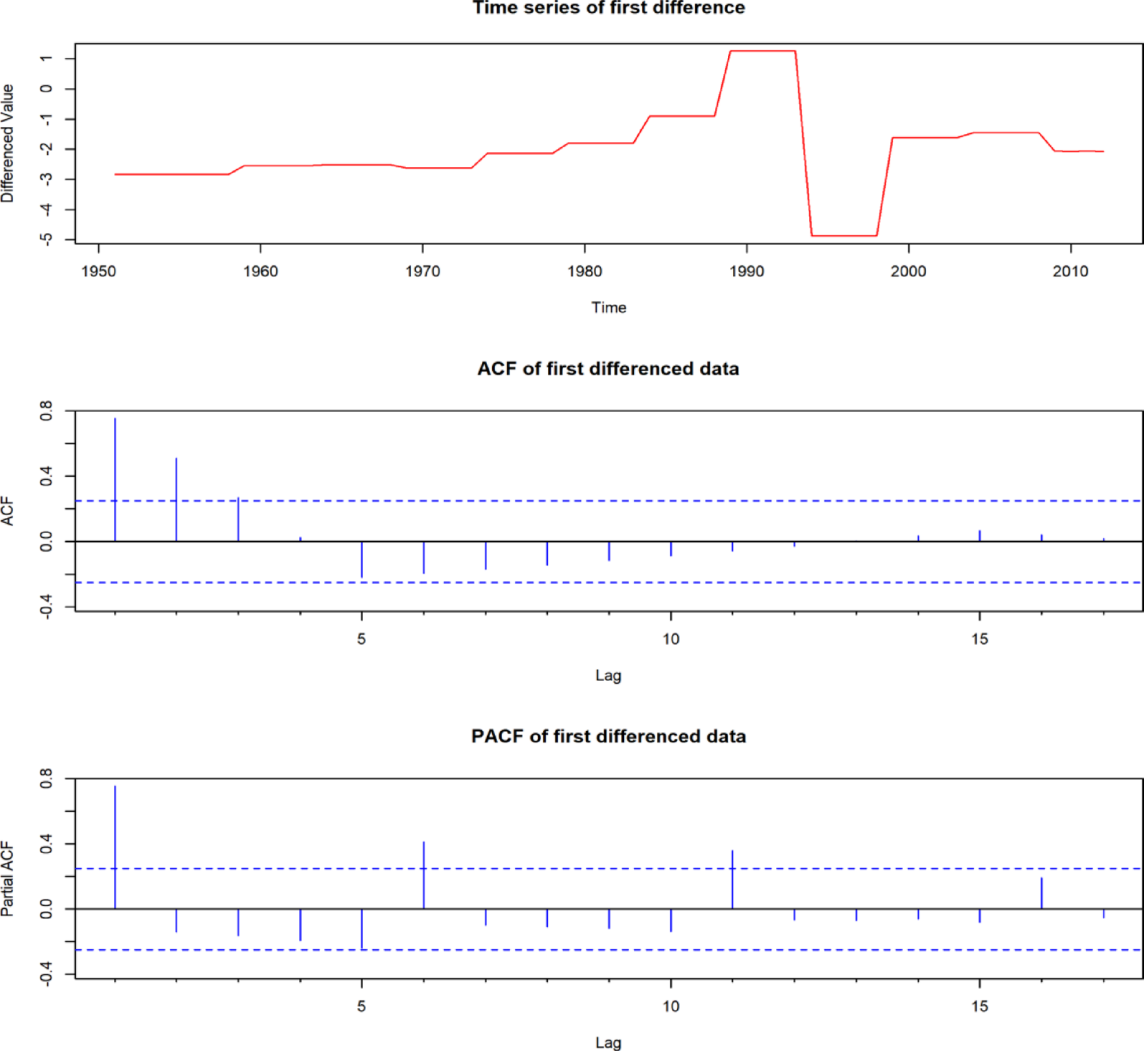
Trend, ACF, and PACF plots for the first differencing infant mortality rate data

The infant mortality rate data is stationary since Augmented Dickey-Fuller and Phillips-Perron have p-values less than 0.05, then the modelling process can begin. The Trend, ACF, and PACF plots are illustrated in Fig. 4.

### ARIMA model identification

This section involves setting the order of (p, d, q) of the model’s AR and MA components in order to capture the data’s silent dynamic properties. It attempts to determine whether the data is stationary or nonstationary, as well as the order of differencing d that causes the time series to be stationary. It mostly results in the usage of graphical approaches. To identify the model, we examined annual data from 1950 to 2022. If the graph of ACF of time series values either cuts off or dies down pretty rapidly, the data is deemed steady; if the graph of ACF quiets down exceedingly slowly, the data is called nonstationary. The time series of the first difference appears to be stationary in mean and variance during the differencing process, implying that an ARIMA (p, 1, q) model is for the time series of infant mortality rate data. Looking at the ACF and PACF of our differencing data, it seems that the p-value from PACF is (1) while the significant lag at 3 in the ACF suggests a Moving Average (MA) order of q = 3. The Augmented Dickey Fuller (ADF) test yielded a p-value of 0.03018 indicating that the infant mortality rate data is stationary at the first difference. To avoid bias, we examined ARIMA (p,1, q) model as well as utilizing R’s auto.arima() function, which selects the ARIMA (1,1,3), because the ndiffs() tool also reported that the difference is 1.

### ARIMA model estimation and selection

This section involves the estimation of parameters of ARIMA model using identification process and proceeds to the selection of the model using information criteria.

From Table 4 the log-likelihood of ARIMA (1, 1, 3) is −80.46, which is the maximum. AIC value is 170.92 and BIC value is 181.55,It has a good fit. For that reason, the model is selected as ARIMA (1, 1, 3). The equation is as follows:

**Table 4:** Parameter estimates for the ARIMA model, their likelihood function and information criterions

ARIMA (p, d, q)	Parameters	Estimate	SE	Log-Likelihood	AIC	BIC
**(1, 1, 3)**	ar1​	0.7756	0.0835	**−80.46**	**170.92**	**181.55**
	ma1​	0.0091	0.0939			
	ma2	0.5503	0.0938			
	ma3​	0.5522	0.1059			
**(1, 1, 0)**	ar1​	0.9255	0.0444	−84.47	172.93	187.19
**(0, 1, 3)**	ma1​	1.4657	0.1145	−96.53	201.53	209.58
	ma2​	0.8901	0.1391			
	ma3​	0.0003	0.0928			

Bold values indicate the optimal model parameters and the best performing model with the lowest error metric

$$\begin{aligned} {X}_{t} & =0.7756{X}_{t-1}+0.0091{\epsilon}_{t-1} \\ & \quad +0.5503{\epsilon}_{t-2}+0.5522{\epsilon}_{t-3}+\epsilon \; t \end{aligned}$$

### Model adequacy checking

As shown in Table 4, the log-likelihood of ARIMA (1,1,3) is −80.46. The AIC value is 170.92, and the BIC is 181.55, indicating a good fit. Consequently, ARIMA (1,1,3) was selected. The Ljung-Box test yielded a p-value of 0.659 in Table 5, confirming that the residuals were random and resembled white noise.

**Table 5:** Ljung box test for ARIMA (1,1,3) model

Test type	X-squared (*x*^2^)	Df	p-value
Ljung box test	16.907	20	0.659

This above Table 6 indicates the accuracy of ARIMA (1,1,3), ARIMA (1,1,0), and ARIMA (0,1,3) model.

**Table 6:** Accuracy of ARIMA (1,1,3), ARIMA (1,1,0), and ARIMA (0,1,3) model

Adequacy measure	ME	RMSE	MAE	MPE	MAPE	MASE
**Models**						
ARIMA (1,1,3)	−0.2122	0.8450	0.5236	−0.1534	0.41177	0.2328398
ARIMA (1,1,0)	−0.14906	0.9232	0.3694	−0.1050	0.29293	0.16424
ARIMA (0,1,3)	−0.60160	1.10257	0.8499	−0.4473	0.64505	0.37791

Beyond basic error metrics, the advanced evaluation criteria confirmed the robustness of the models. Both the ARIMA and ANN models demonstrated exceptional predictive capability. The ARIMA model yielded a Willmott’s Index (WI) of 0.985, an Absolute Percentage Bias (APB) of 1.09%, and an Explained Variance Score (EVS) of 0.952. The ANN model yielded similarly robust results (WI = 0.990, APB = 1.25%, EVS = 0.956). Conversely, the Hybrid model underperformed across all advanced metrics, particularly exhibiting a higher absolute bias (WI = 0.896, APB = 3.94%, EVS = 0.944).

### Forecasting with ARIMA model

In this section, we used annual data for infant mortality rate of Somalia from the year 1950 to 2022 to build the model. The ARIMA model is given by:

$$\begin{aligned}\phi\left(B\right) \Delta^\wedge d \; X= \left(B\right) \xi \end{aligned}$$

For infant mortality rate data model, the values of *p* = 1, d = 1, and q = 3. Hence our model becomes:

$$\begin{aligned}\phi\left(B\right) \Delta^\wedge 1 \; X= \left(\textit{B}\right) \xi \end{aligned}$$

Using R statistical software, we obtain the model parameters as follows: $\:\phi\:1=0.504$, $\:\theta\:1=0.0594$, $\:\theta\:2=-0.5527$, $\:\theta\:3=0.5879$.

$$\begin{aligned}Xt & =0.7756Xt-1+0.0091\epsilon\:t-1+0.5503\epsilon\:t\\ & \quad-2+0.5522\epsilon\:t-3+\epsilon\:t \end{aligned}$$

**Fig. 5:**
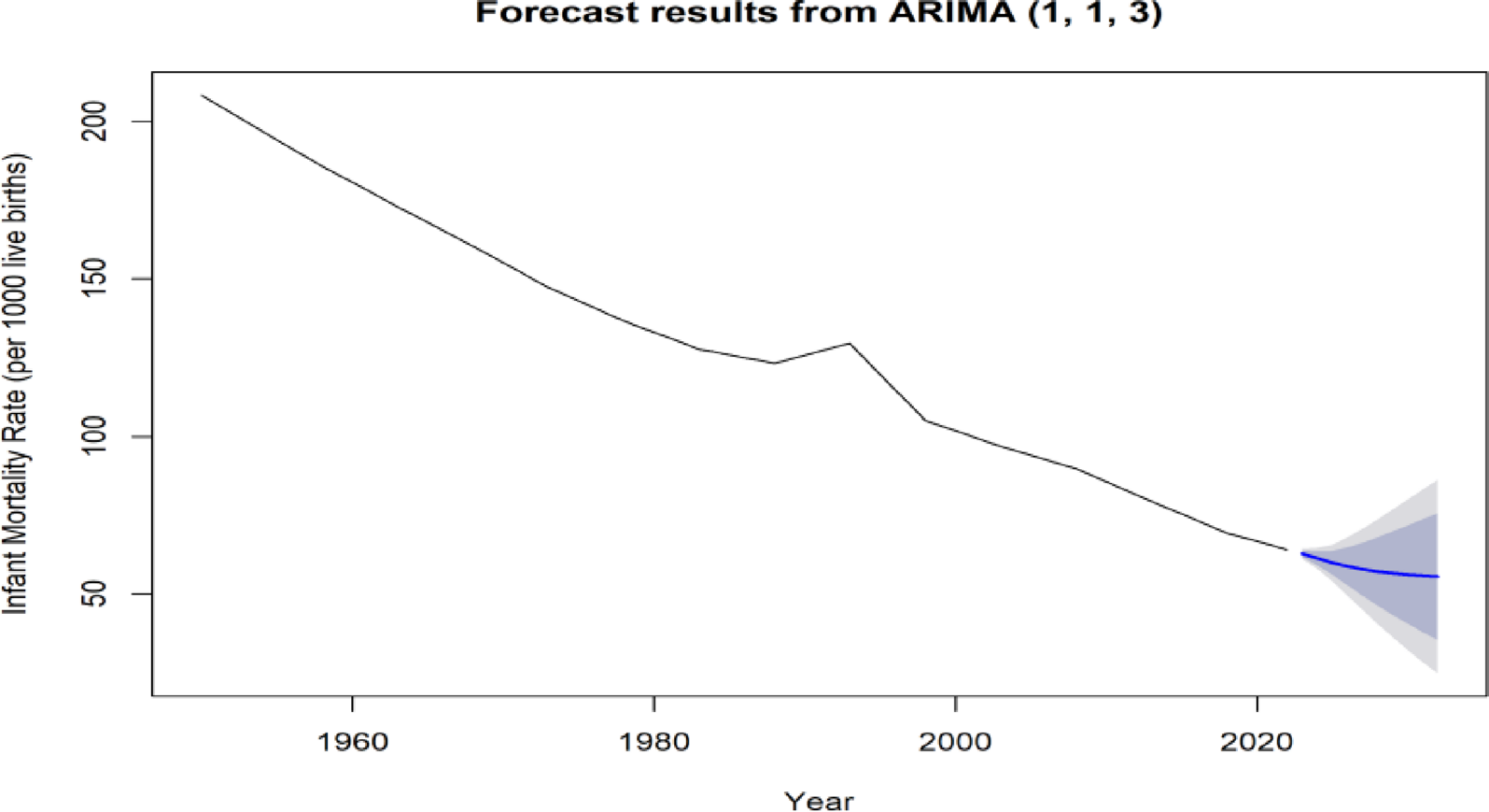
Forecast results from ARIMA (1, 1, 3)

With the equation above, we have used it to forecast the infant mortality rate data from 2023 to 2032 as shown in Table 7. The values for the forecast are represented graphically as shown in Fig. 5 at a confidence interval of 95%. The actual data did not match the predicted data perfectly, but fell within the predicted 95% confidence interval with their forecast point and their lower and upper limits.

**Table 7:** Forecast results from ARIMA (1, 1, 3) infant mortality rate in Somalia from 2023 to 2032

Year	Point forecast	Lower 95% CI	Upper 95% CI
2023	59.02	39.32	78.72
2024	56.95	36.00	77.90
2025	54.88	32.74	77.01
2026	52.81	29.55	76.07
2027	50.73	26.40	75.07
2028	48.66	23.31	74.02
2029	46.59	20.25	72.93
2030	44.52	17.22	71.81
2031	42.45	14.23	70.66
2032	40.37	11.27	69.48

### Artificial neural network

**Fig. 6:**
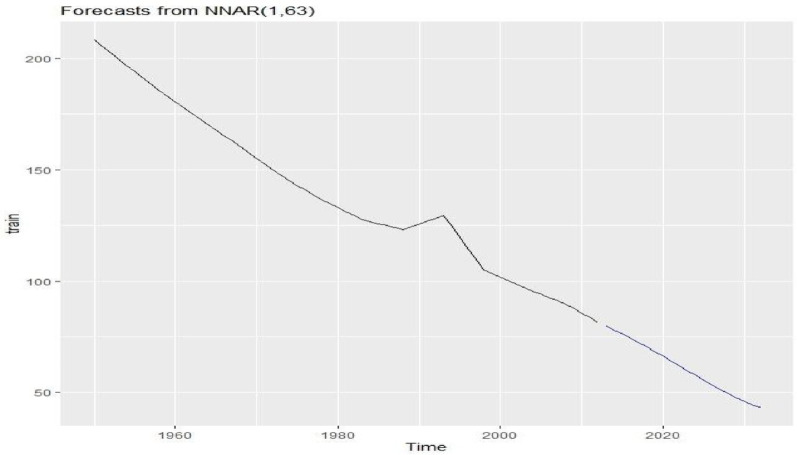
Forecast from ANN (1,3,1) [73] model

The complete dataset was modelled using an Artificial Neural Network (ANN) that employed the Neural Network Autoregressive function to produce an NNAR (p, P,k) [m] model. The optimal lag parameter, p, and the number of nodes in the hidden layer, k, were automatically selected while *P* = 1 by default. In addition, a decay parameter of 0.001 and a maximum iteration of 200 were pre-set for the model to help restrict the weights from becoming too large and ensure that the model can test various models until the model that yielding the lowest RMSE was identified. The optimal NNAR model generated was NNAR (1,3,1) [73]. The plot of the point forecast values from the NNAR (1,3,1) [73] model on the general data set were presented in Fig. 6. Table 9 presents the model’s accuracy measures. The model performance on the overall data set appears poor although the MAPE values below 50% are reasonable.

This above Table 8 deploys Forecast results from ANN (1,3,1) [73] infant mortality rate in Somalia from 2023 to 2032.

**Table 8:** Forecast results from ANN (1,3,1) [73] infant mortality rate in Somalia from 2023 to-2032

Year	Point forecast
2023	59.76
2024	57.60
2025	55.46
2026	53.38
2027	51.37
2028	49.46
2029	47.66
2030	45.99
2031	44.45
2032	43.06

In Table 9 the forecasting error has been calculated as the RMSE for the filter time series. Its value is 1.04, which indicates that the forecasting power of ANN is reliable and gives statistically significant results in this study. This means that ANN provides a good forecasting model that can be relied upon in any similar time series.

**Table 9:** Accuracy of ANN (1,3,1) [73] model

	ME	RMSE	MAE	MPE	MAPE	ACF1
**Test set**	−0.1084717	1.03695	0.8942387	−0.08286962	1.294975	0.6273457

### Hybrid (ARIMA-ANN) model

**Fig. 7:**
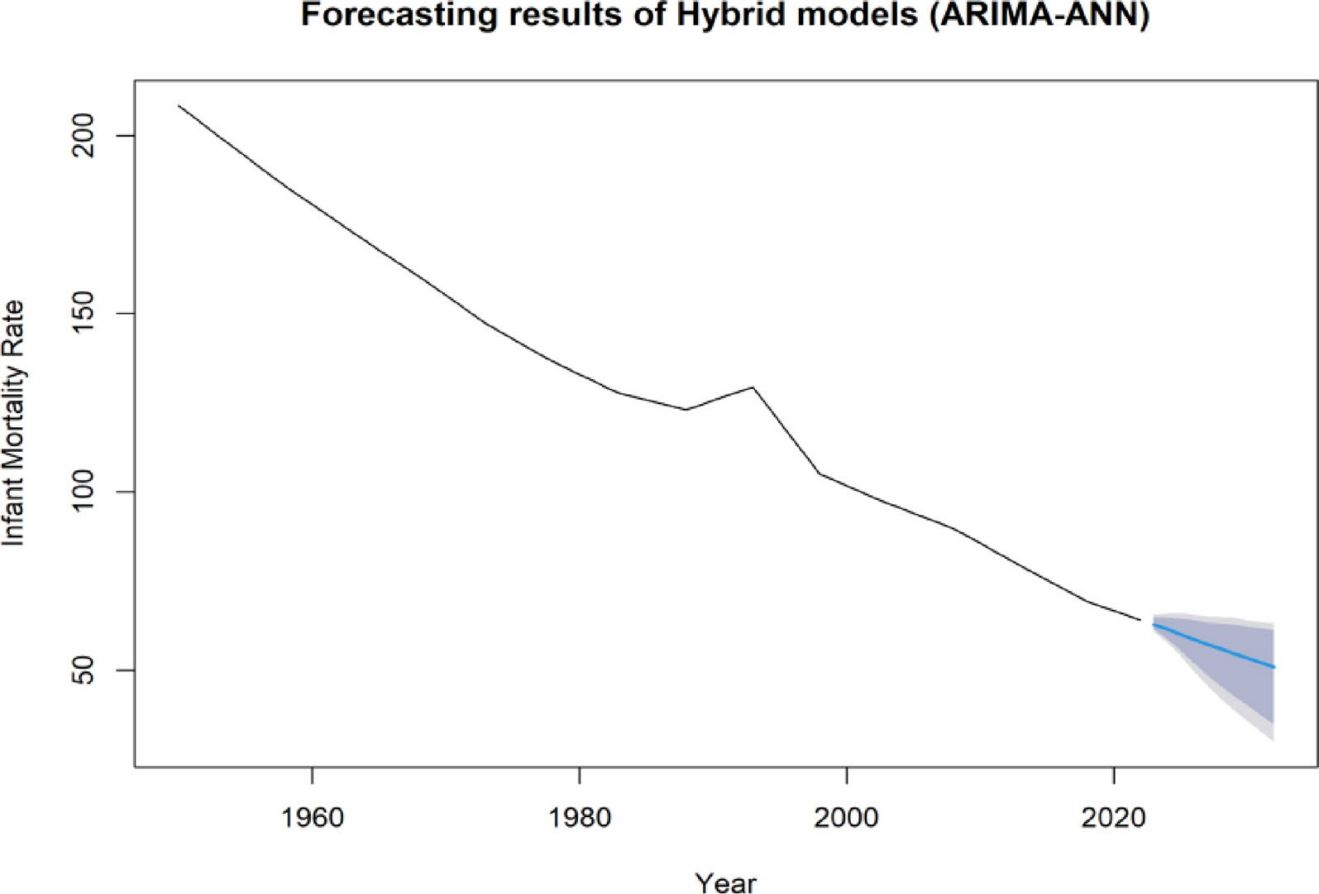
Forecasting results of Hybrid models (ARIMA-ANN)

Generally, a time series can be observed as having linear and nonlinear components, as shown in the equation:

$$\:{Y}_{t}={\mathrm{I}}_{t}+{n}_{t}$$

where lt and nt are the linear (from the ARIMA model) and nonlinear (ANN fitted ARIMA model residuals) components respectively. Residuals from the ARIMA model are fitted with the ANN model (see Fig. 7).

Residuals from the ARIMA model are fitted with the ANN model. The Hybrid model yielded an RMSE of 3.01. This indicates that for this specific dataset, the Hybrid approach did not improve predictive accuracy compared to the standalone ARIMA model. *The forecasting results are shown in* Table 10*and “The hybrid model accuracy is shown in* Table 11.

**Table 10:** Forecasting results of Hybrid models (ANN-ARIMA)

Year	Point forecast	95% CI lower	95% CI upper
2023	66.65	39.32	80.27
2024	65.43	36.00	80.72
2025	64.22	32.74	80.17
2026	63.03	29.55	80.47
2027	61.85	26.40	80.04
2028	60.69	23.31	79.19
2029	59.54	20.25	78.97
2030	58.40	17.22	78.58
2031	57.27	14.23	78.16
2032	56.14	11.27	78.15

**Table 11:** Hybrid (ARIMA-ANN) model

	ME	RMSE	MAE	MPE	MAPE	ACF1
**Test set**	−2.777343	3.012044	2.777343	−4.035316	4.035316	0.7045944

## Model comparison

### Forecasting results

Forecast values for 2023–2032 are fully detailed *in* Table 12.

**Table 12:** Comparison of models performance in forecasting infant mortality rate

Year	ARIMA	ANN	ARIMA-ANN
2023	59.02	59.76	66.65
2024	56.95	57.60	65.43
2025	54.88	55.46	64.22
2026	52.81	53.38	63.03
2027	50.73	51.37	61.85
2028	48.66	49.46	60.69
2029	46.59	47.66	59.54
2030	44.52	45.99	58.40
2031	42.45	44.45	57.27
2032	40.37	43.06	56.14

### **M**odel adequacy checking

A comparison has been made between the results obtained from applying ARIMA, ANN and their hybrid models.

### **S**ymmetric mean absolute percentage error

Symmetric Mean Absolute Percentage Error (Smape) is a metric that is used to evaluate the accuracy of the forecasting model. As Table 13 indicates Smape of the ARIMA model is 0.01206882 so; this ARIMA model has least error.It fails in 1.2% error. Smape of the ANN model is 0.0129901 so, this ANN model has least error.It fails in 1.29% error. Smape of the hybrid (ARIMA-ANN) model is 0.03939401 so, this (ARIMA-ANN) model has least error since it fails in 3.93% as error.The study can conclude from the above discussion that the results of ARIMA model are much better than those of the ANN model, and hybrid (ANN-ARIMA) models, which made the forecast more efficient with less RMSE and smape.

**Table 13:** Comparison of RMSE and Smape of ANN, ARIMA and hybrid (ANN-ARIMA) models

Models	ARIMA model	ANN model	Hybrid (ANN-ARIMA)
**RMSE**	0.8450687	1.036957	3.012044
**SMAPE**	**0.01206882**	**0.0129901**	**0.03939401**

Bold values indicate the optimal model parameters and the best performing model with the lowest error metric

## Discussion

This section discusses the study’s findings. All three models were able to forecast the infant mortality rate; however, the ARIMA model offered better forecasting performance compared to the ANN and hybrid ARIMA-ANN models.

To identify the appropriate ARIMA model, it is necessary to identify the order of differencing needed to stationarize the time series of the infant mortality rate in Somalia. Several tests were considered to ensure the stationarity of the time-series, such as the ADF and KPSS tests, which found that the most suitable order of differencing was the first difference. To select the suitable order of the ARIMA model (p, q), the study performed procedures and tests for testing the significance of coefficients and the residuals, such as finding ACF, PACF, and the Ljung-Box test. To estimate the best ARIMA model, the study used two criteria: the Akaike’s information criterion (AIC) and the Bayesian information criterion (BIC). The suitable order of the best ARIMA model was ARIMA(1,1,3). The study examined the results of this model and found that it produces good forecasts which come close to the actual values. The forecast limits contain all the actual values, though the intervals are wide. The study observed that the Root Mean Square Error (RMSE) of the ARIMA model is small (0.845). These results align with other literature; for instance, a study analyzing the IMR in Nigeria from 1964 to 2018 using ARIMA modelling selected ARIMA (1,1,1) as the best model, revealing a projected reduction of 30% in the IMR by 2030 [13].

To ensure the robustness of our predictions, validation was conducted using alternative forecasting horizons (h = 5 and h = 10 years). Across both the short-term and medium-term horizons, the ARIMA model consistently maintained the lowest error metrics. Furthermore, while the ARIMA forecast accurately captures the trajectory of the test data, the 80% and 95% confidence intervals remain relatively wide. This is not a failure of the model, but rather a mathematical reflection of the extreme historical volatility in the training data—specifically the massive structural shocks to the IMR caused by the state collapse in the 1990s.

Regarding the ANN model, figures such as Fig. 6; Table 9 illustrate a reasonable level of forecasting accuracy. The study found that the minimum RMSE value for the ANN time series was 1.037. These findings are relevant to prior studies; for example, an ANN approach was applied to analyze IMR in Ethiopia using annual data from 1966 to 2020. The evaluation criteria indicated that the ANN (12, 12, 1) model was stable, predicting IMR to be around 34/1000 live births per year in the out-of-sample period [14].

In contrast, the hybrid model (ARIMA-ANN) yielded an RMSE value of 3.012, which was the highest root mean square error among the approaches, placing its forecasting performance below both the standalone ARIMA and ANN models.

**Model performance and the principle of parsimony** To determine if the differences in predictive accuracy between the models were statistically significant, a Diebold-Mariano test was conducted. The test yielded a p-value of 1.000 when comparing ARIMA to ANN, revealing no statistically significant difference in their forecast accuracy over the 10-year testing horizon. Because both models are statistically indistinguishable in their predictive power for this specific dataset, the principle of parsimony dictates that the simpler, linear ARIMA model is the scientifically preferred choice for forecasting Somalia’s IMR.

Interestingly, the Hybrid ARIMA-ANN model did not outperform the standalone ARIMA model. While hybrid models are theoretically designed to capture both linear and non-linear patterns, their efficacy depends heavily on the presence of complex, non-linear residuals. In the case of Somalia’s Infant Mortality Rate, the post-2000 era is characterized by a strong, relatively stable downward linear trend driven by consistent international aid and the gradual re-establishment of basic maternal health programs. Consequently, the ARIMA model successfully captured the vast majority of the variance. The residuals fed into the ANN component of the hybrid model lacked sufficient non-linear signal, causing the neural network to fit noise, thereby degrading the overall forecast accuracy compared to the pure ARIMA approach.

Contextualizing these mathematical results within the historical data, the time plot of the infant mortality rate (Fig. 1) shows a downward trend, indicating that the IMR in Somalia has been constantly decreasing from 208 in 1950 to 64 in 2022. Within these years, there is an approximate triangle movement between 1988 and 1999—aligning with the structural breaks of the civil war—where the infant mortality rate fluctuated significantly between 123 and 103.

The observed decline in IMR in Somalia aligns with global trends, although regional disparities persist [15]. The superiority of the ARIMA model in this context highlights its effectiveness for forecasting health indicators in settings with limited long-term, high-quality data [16]. The demonstrated superiority of the ARIMA model is attributed to the relatively stable, consistent downward trend of the infant mortality data over the observation period following the conflict years. ARIMA models are particularly efficient at handling such linear trajectories through differencing (d = 1). The complexity of neural networks was not required to capture this specific trend, further adhering to the principle of parsimony in statistical modelling. While the ANN and hybrid models showed promise, their higher RMSE values suggest potential overfitting to the training data or a lack of robustness to variations in this specific time series [17].

The consistent downward trend predicted by the ARIMA model provides a valuable tool for public health officials to anticipate future needs and allocate resources efficiently for maternal and child health programs [18]. However, it is crucial to acknowledge that while statistical models offer powerful predictive capabilities, they should be complemented by contextual knowledge and expert judgment for policy implementation [19]. Future research could explore incorporating exogenous variables, such as climate change indicators or conflict intensity, into these models to further enhance forecasting accuracy, particularly in fragile contexts like Somalia [20]. Ultimately, these results suggest that one of the key contributions of our paper is the rigorous evaluation of the utility of machine learning approaches versus classical statistical methods in data-scarce, conflict-affected environments. These findings also align with recent advancements in epidemiological forecasting, which increasingly demonstrate the efficacy of both standalone and hybrid machine learning architectures—such as advanced Artificial Neural Networks and filtering techniques—in predicting complex public health and mortality trends across varying horizons [21–24].

Based on the expanded evaluation criteria outlined in Table 14, both the ARIMA and ANN models demonstrated excellent predictive agreement, significantly outperforming the hybrid ARIMA-ANN model. To ensure a robust assessment of forecasting accuracy, this study incorporated advanced criteria including Willmott’s Index of Agreement, Absolute Percentage Bias, and Explained Variance Score, aligning with established methodologies in recent predictive modeling literature [25–29]. While the ANN model achieved slightly higher scores in Willmott’s Index (WI = 0.990) and Explained Variance (EVS = 0.956), the ARIMA model maintained the lowest Root Mean Square Error (RMSE = 0.845) and the lowest Absolute Percentage Bias (APB = 1.09%). Conversely, the hybrid model performed notably worse across all advanced metrics (WI = 0.896, APB = 3.94%, EVS = 0.944). The study concludes that, due to its superior performance in minimizing absolute error and bias, the ARIMA model is the most efficient and scientifically robust choice for forecasting the infant mortality rate in this context.

**Table 14:** Comparison of performance metrics (RMSE, WI, APB, and EVS) of ARIMA, ANN, and hybrid ARIMA-ANN models

Models	RMSE	Willmott’s index (WI)	Absolute Percentage Bias (APB)	Explained Variance Score (EVS)
**ARIMA model**	0.845	0.985	1.09%	0.952
**ANN model**	1.037	0.990	1.25%	0.956
**Hybrid (ARIMA-ANN)**	3.012	0.896	3.94%	0.944

### Limitations

This study is subject to several limitations that should be considered when interpreting its findings. First, the reliability of the dataset is a potential concern; although sourced from the World Bank, the data for Somalia, particularly during periods of civil conflict and governmental instability, may be subject to estimation errors and inconsistencies. Additionally, The analysis was univariate in nature, relying solely on past values of IMR. This is a significant limitation as infant mortality is multifaceted and sensitive to exogenous shocks. The current models do not account for external covariates such as GDP per capita, conflict intensity indices, rainfall patterns (droughts), or healthcare expenditure, which are highly relevant in the Somali context. Incorporating these multivariate factors could potentially improve model robustness. The models did not incorporate other significant determinants such as healthcare expenditure, maternal education levels, or political stability, which, if included, could potentially yield a more robust forecasting model. Moreover, the scope of the models was confined to ARIMA, a specific ANN architecture, and one hybrid combination; other time-series techniques, such as Holt-Winter’s Exponential Smoothing or more advanced deep learning models, were not evaluated and might offer different performance outcomes. Finally, the findings regarding the superiority of the ARIMA (1,1,3) model possess limited generalizability, as the results are specific to the unique historical pattern of Somalia’s IMR data and may not be applicable for forecasting IMR in other countries or for modeling other health indicators with different statistical properties.

## Conclusion

The modeling of time series data for infant mortality in Somalia shows a consistent downward trend. The results indicate that the ARIMA model outperformed the machine learning model (ANN) and the hybrid (ARIMA-ANN) model across all performance measures. Consequently, ARIMA provides a reliable framework for forecasting future trends. The results project a further reduction in the infant mortality rate in Somalia in the coming decade.

## Data Availability

The datasets generated and/or analyzed during the current study are available in the Somalia microdata repository, which can be accessed at: [https://www.macrotrends.net/countries/SOM/somalia/infant-mortality-rate] (https:/www.google.com/url?sa=E&q=https%3A%2F%2Fwww.macrotrends.net%2Fcountries%2FSOM%2Fsomalia%2Finfant-mortality-rate). The data that support the findings of this study are available on request from the corresponding author.
